# The Mechanism of Peach Gum Polysaccharide Preventing UVB-Induced Skin Photoaging by Regulating Matrix Metalloproteinanse and Oxidative Factors

**DOI:** 10.3390/molecules28104104

**Published:** 2023-05-15

**Authors:** Min Yang, Liang Tao, Zilin Wang, Lingfei Li, Junyi Luo, Kuannu Pai, Weitong Li, Cunchao Zhao, Jun Sheng, Yang Tian

**Affiliations:** 1 College of Food Science and Technology, Yunnan Agricultural University, Kunming 650201, Chinataowuliang@163.com (L.T.);; 2 Engineering Research Center of Development and Utilization of Food and Drug Homologous Resources, Ministry of Education, Yunnan Agricultural University, Kunming 650201, China; 3 Key Laboratory of Precision Nutrition and Personalized Food Manufacturing, Ministry of Education, Yunnan Agricultural University, Kunming 650201, China; 4 National Research and Development Professional Center for Moringa Processing Technology, Yunnan Agricultural University, Kunming 650201, China; 5 PuEr University, Puer 665000, China

**Keywords:** peach gum polysaccharide, skin photoaging, oxidative factors, matrix metalloproteinase, antioxidant functional food

## Abstract

Exposure to ultraviolet light can cause oxidative damage and accelerate skin aging and is one of the main causes of skin aging. Peach gum polysaccharide (PG) is a natural edible plant component that has many biological activities, such as regulating blood glucose and blood lipids and improving colitis, as well as antioxidant and anticancer properties. However, there are few reports on the antiphotoaging effect of peach gum polysaccharide. Therefore, in this paper, we study the basic composition of the raw material peach gum polysaccharide and its ability to improve UVB-induced skin photoaging damage in vivo and in vitro. The results show that peach gum polysaccharide is mainly composed of mannose, glucuronic acid, galactose, xylose, and arabinose, and its molecular weight (M*w*) is 4.10 × 10^6^ g/mol. The results of the in vitro cell experiments show that PG could significantly alleviate UVB-induced apoptosis of human skin keratinocytes, promote cell growth repair, reduce the expression of intracellular oxidative factors and matrix metal collagenase, and improve the extent of oxidative stress repair. Moreover, the results from the in vivo animal experiments showed that PG could not only effectively improve the phenotype of UVB-induced photoaged skin in model mice but also significantly improve their oxidative stress status, regulate the contents of ROS and the levels of SOD and CAT, and repair the oxidative skin damage induced by UVB in vivo. In addition, PG improved UVB-induced photoaging-mediated collagen degradation in mice by inhibiting the secretion of matrix metalloproteinases. The above results indicate that peach gum polysaccharide has the ability to repair UVB-induced photoaging and may be used as a potential drug and antioxidant functional food to resist photoaging in the future.

## 1. Introduction

The skin is the most widely distributed and complex organ in the human body. It is in direct contact with the external environment and protects the internal structures of the body from it [1]. Skin aging is a complex physiological phenomenon caused by both internal factors and external factors [2,3]. Ultraviolet (UV) radiation is harmful to human skin. Long-term exposure to UV radiation can lead to sunburn, immunosuppression, cancer, and skin photoaging [4,5]. UV radiation can be divided into UVA (320–400 nm), UVB (280–320 nm), and UVC (200–280 nm) [6]. Notably, UVB causes significant biological damage to the skin, significantly affects the growth and development of the epidermis and causes premature skin aging [7,8]. Skin photoaging is a common skin condition with the common clinical manifestations of the loss of skin elasticity, epidermal atrophy, deep wrinkles, and abnormal pigmentation, which can lead to cell apoptosis or carcinogenesis [9,10]. Moreover, the epidermis is the main protector of the body against environmental damage. Most of the epidermis is composed of keratinocytes and melanocytes [11]. Melanocytes can synthesize and store melanin and protect the skin from harmful stimuli such as ultraviolet rays. However, many of the clinical manifestations of photoaging are caused by excessive production and accumulation of melanin in human skin. Ultraviolet radiation can directly affect melanocytes, regulate the expression of melanogenic enzymes, and cause skin photoaging [12]. Some studies have shown that natural plants play an important role in skin whitening by inhibiting tyrosinase expression and alleviating the excessive accumulation of melanin caused by ultraviolet light [13].

Studies have shown that UVB can induce the production of matrix metalloproteinases (MMPs) in the skin. MMPs have a wide range of substrate specificities and play important roles in angiogenesis, tumor invasion, and photoaging [14]. Moreover, MMPs can degrade skin-related collagen and other extracellular matrix proteins. In particular, MMP-1 is a major component of the MMP interstitial collagenase subfamily and is also associated with connective tissue damage, such as damage to type I and type III collagen. Therefore, MMP-1 is considered to be the main marker of skin aging. Inhibition of MMP-1 expression is considered to be a good strategy to prevent wrinkle formation and photoaging [15,16]. MMP-3 is a matrix hydrolase mainly distributed in the extracellular matrix that can degrade denatured collagen [17].

In addition, repeated exposure to UVB irradiation can produce a large amount of reactive oxygen species (ROS) in skin tissue. Excessive ROS can damage biological macromolecules such as proteins and nucleic acids in cells, cause cell aging, and even lead to apoptosis [18,19]. Moreover, a large number of studies have shown that an abnormal increase in the activity of oxidative factors in vivo is a significant cause of skin aging [14,20]. Therefore, oxidative stress is considered to be an important mechanism of aging in the human body, and inhibition of oxidation reactions enhances the antiaging effect to extend life expectancy [21,22]. In addition, ROS is one of the important factors leading to wrinkle formation. ROS-induced oxidative stress promotes the production of pro-inflammatory tumor necrosis factor and interleukins. ROS as a pro-inflammatory factor may cause MMPs’ activation in the skin, and activated matrix metalloproteinases can degrade cell matrix components, resulting in loss of skin elasticity and water [23,24]. Some studies have shown that *Isatis indigotica* leaves have the effect of scavenging DPPH and ABTS free radicals and have shown excellent anti-wrinkle potential effect [25].

Peach gum is a resin component secreted from the bark of peach or mountain peach trees that is rich in resources and widely used in food and medicine [26]. Peach gum polysaccharide (PG) is one of the main active components of peach gum. Peach gum polysaccharide usually contains complex polysaccharides and a large number of monosaccharides, as well as glycosidic bonds, mostly with highly branched structures [27,28]. A large number of studies have shown that peach gum polysaccharide has antiaging, blood lipid-lowering, immunity-improving, antitumor, free radical-scavenging, blood sugar-lowering, and other effects [29,30,31]. Studies have shown that natural plant-derived polysaccharides have good antioxidant activity, as they can scavenge excessive free radicals produced in the body and relieve oxidative damage [32,33,34]. Some polysaccharides can also play significant roles against skin photoaging in vitro and in vivo by improving antioxidant activity or immune function [35,36]. However, the preventive effect and molecular mechanism of peach gum polysaccharide on photoaging are not fully understood. Therefore, in this study, we use HaCaT human keratinocytes and hairless mice to detect the effect of peach gum polysaccharide on UVB-induced skin aging. The results show that peach gum polysaccharide attenuates UVB-induced photoaging damage in vivo and in vitro by inhibiting the expression of matrix metalloproteinases and antioxidant factors in a UVB-induced HaCaT cell photoaging model and animal model. The results of this study provide a new idea for the preparation of peach gum polysaccharide and its application in antiphotoaging functional foods and drugs.

## 2. Results

### 2.1. Physicochemical Properties of Peach Gum Polysaccharide

As shown in Table 1 and Table 2, peach gum polysaccharide is mainly composed of mannose, glucuronic acid, galactose, galacturonic acid, xylose, and arabinose, and the molecular weight (M*w*) is 4.10 × 10^6^ g/mol (Table 1). The content of uronic acid in peach gum polysaccharide is high, and the proportions of galacturonic acid and glucuronic acid are large (Table 2 and Figure S1). The study found that the activity of polysaccharides may be determined by molecular weight and chemical structure [37]. Studies have shown that high molecular weight polysaccharides such as fucoidan have strong antioxidant and antiphotoaging activities and may directly scavenge free radicals and regulate the intestinal flora [38]. Some studies have also shown that polysaccharides with high galacturonic acid content have significant antioxidant activity [39].

### 2.2. Effect of Peach Gum Polysaccharide on HaCaT Cells

**Figure 1:**
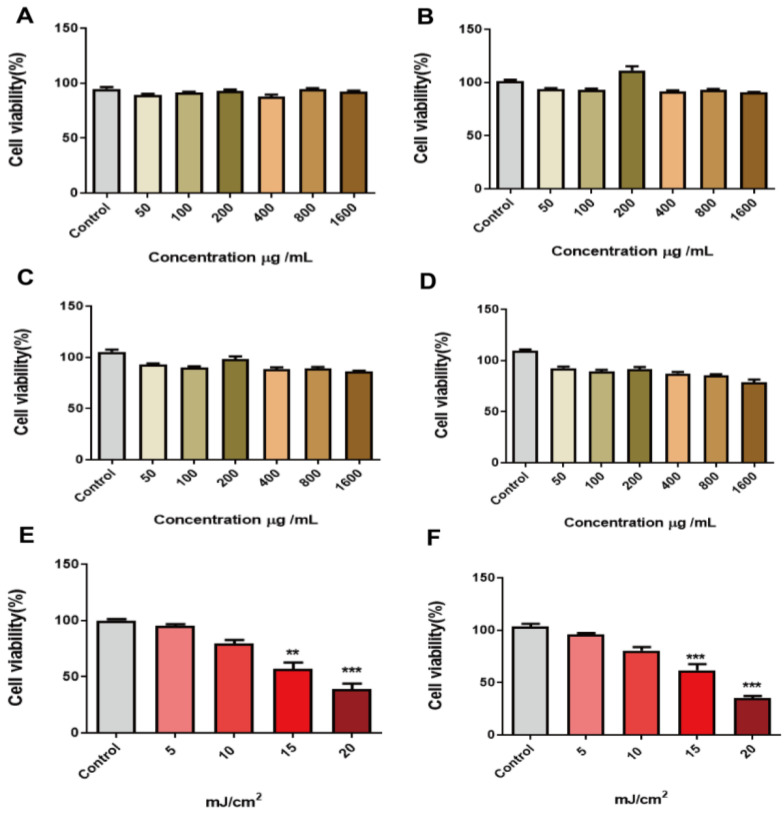
Peach gum polysaccharide promoted the proliferation of HaCaT cells. (**A**) Promotion of HaCaT cell proliferation by different concentrations of peach gum polysaccharide after 12 h. (**B**) Promotion of HaCaT cell proliferation by different concentrations of peach gum polysaccharide after 24 h. (**C**) Promotion of HaCaT cell proliferation by different concentrations of peach gum polysaccharide after 48 h. (**D**) Promotion of HaCaT cell proliferation by different concentrations of peach gum polysaccharide after 72 h. (**E**) Alleviation of HaCaT cell proliferation by different UVB irradiation doses after pretreatment with polysaccharides for 12 h. (**F**) Alleviation of HaCaT cell proliferation by different UVB irradiation doses after pretreatment with polysaccharides for 24 h. Data are expressed as the mean ± SEM from three independent experiments (*n* = 3). ** *p* < 0.01, *** *p* < 0.001 vs. control group.

To detect the toxicity of peach gum polysaccharide for normal HaCaT cells, HaCaT cells were cultured with 50 μg/mL, 100 μg/mL, 200 μg/mL, 400 μg/mL, 800 μg/mL, and 1600 μg/mL peach gum polysaccharide for 12 h, 24 h, 48 h, and 72 h. The MTT assay was used to evaluate the toxicity of the polysaccharide to the cells. The results in Figure 1 show that peach gum polysaccharide at different concentrations (0–1600 μg/mL) did not show toxicity to HaCaT after treatment for 12 h, 24 h, 48 h, and 72 h (Figure 1A–D). Moreover, peach gum polysaccharide showed a significant proliferation effect on the cells, especially when the concentration was 200 μg/mL and the incubation time was 24 h, and the cell viability increased by approximately 1.1 times compared with that in the control group, and when the concentration was 800 μg/mL and the incubation time was 24 h, and the cell viability increased by approximately 0.9 times compared with that in the control group (Figure 1B). A large number of studies have shown that plant polysaccharide components can promote the proliferation of HaCaT cells [35,38], and it has also been reported that peach gum can promote the growth of macrophages [29]. Some research evaluated peach gum polysaccharide in HepG2 and 3T3-L1 cells using the MTT assay. The results clearly showed that peach gum polysaccharide at the range of 0.078–5.00 mg/mL did not decrease cell viability [28]. Therefore, subsequent experiments were carried out with peach gum polysaccharide in the concentration range of 0–800 μg/mL.

### 2.3. UVB Radiation Dose Dependently Reduced the Viability of HaCaT Cells

Ultraviolet radiation, especially UVB radiation, can damage skin cells. The more powerful part of UVB radiation energy acts on the skin surface, which can lead to gene mutations in skin cells and even skin cancer [40,41]. In our study, HaCaT cells were irradiated with UVB at 5, 10, 15, and 20 mJ/cm^2^. After UVB irradiation, HaCaT cells were cultured for 12 h and 24 h. Cell viability was determined by MTT assay. The results showed that, compared with the control group, the cell survival rates were 87%, 76%, 61%, and 45% after culture for 12 h at 5, 10, 15, and 20 mJ/cm^2^ UVB irradiation, respectively (Figure 1E). In contrast, when the cells were cultured for 24 h, the cell viability was 89%, 83%, 65%, and 37%, respectively (Figure 1F). The results showed that the cells recovered after 12 h and 24 h of UVB irradiation. Compared with the control group, cell viability after irradiation decreased significantly (*p* ≤ 0.01, *p* ≤ 0.001). Interestingly, due to the self-recovery ability of the cells, cell viability at 24 h was slightly restored compared with that after 12 h of culture, but the cell viability was far from that of normal cells, indicating that this dose of UVB irradiation can damage HaCaT cells (Figure 1E,F). Previous studies have shown that when the UVB irradiation dose is high, the expression of matrix metalloproteinases and oxidative factors is significantly increased. These conditions can lead to intracellular high levels of oxidative stress, as the keratinocyte barrier is seriously damaged [42,43]. Therefore, the appropriate dose of UVB to establish a UVB-induced photoaging model in HaCaT cells is 15 mJ/cm^2^, and these model cells should be incubated for 24 h after irradiation.

### 2.4. Peach Gum Polysaccharide Protects HaCaT Cells from UVB-Induced Apoptosis

**Figure 2:**
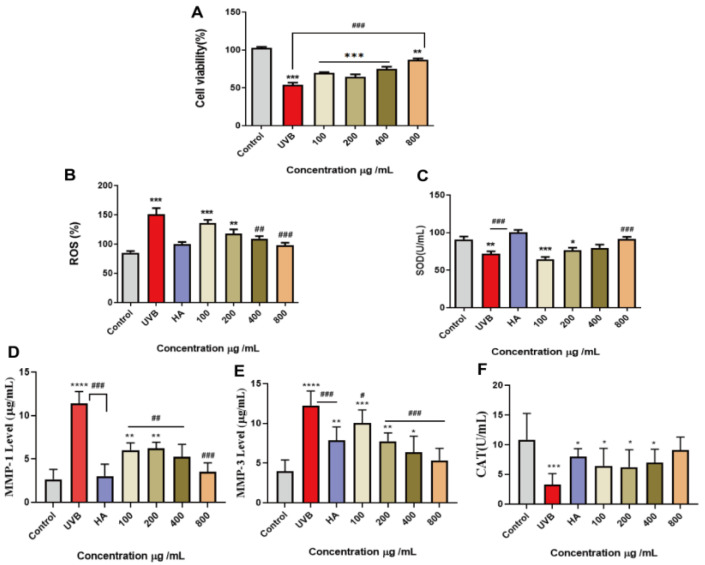
Peach gum polysaccharide restored cellular antioxidant activity and reduced the high expression of MMPs induced by UVB irradiation. (**A**) Different concentrations of peach gum polysaccharide promoted the proliferation of HaCaT cells. (**B**) ROS level in HaCaT cells after UVB irradiation. (**C**) SOD level in HaCaT cells after UVB irradiation. (**D**) MMP-1 level in HaCaT cells after UVB irradiation. (**E**) MMP-3 level in HaCaT cells after UVB irradiation. (**F**) CAT level in HaCaT cells after UVB irradiation. Data are expressed as the mean ± SEM from three independent experiments (*n* = 3). * *p* < 0.05, ** *p* < 0.01, *** *p* < 0.001, **** *p* < 0.0001 vs. control. **^#^** *p* < 0.05, **^##^** *p* < 0.01, **^###^** *p* < 0.001 vs. UVB group.

To further explore the effect of peach gum polysaccharide on UVB-induced skin photoaging repair, we investigated the effects of 100 μg/mL, 200 μg/mL, 400 μg/mL, and 800 μg/mL peach gum polysaccharide on UVB-induced photoaging in the HaCaT cell model. The results are shown in Figure 2A. After HaCaT cells were irradiated with 15 mJ/cm^2^ UVB, compared with the control group, the cell viability in the UVB model group was significantly reduced (approximately 53% of that in the control group). However, when cells were treated with different concentrations of peach gum polysaccharide and then irradiated with UVB, the results showed that peach gum polysaccharide could improve cell viability, and the effect was positively correlated with the dose. When the concentration of peach gum polysaccharide was 400 μg/mL and 800 μg/mL, cell viability increased significantly. Moreover, cell viability in the groups with different doses of peach gum polysaccharide was significantly higher than that in the UVB model group. In summary, UVB irradiation reduced the viability of HaCaT cells, while peach gum polysaccharide treatment promoted HaCaT cell growth after irradiation. After pretreatment with higher concentrations of peach gum polysaccharide, cell viability was significantly higher than that in the UVB model group, indicating that peach gum polysaccharide may promote cell proliferation.

### 2.5. Effect of Peach Gum Polysaccharide on the Production of Oxidative Factors in HaCaT Cells

Cells are the basic structures of organisms, and the aging process in organisms is often accompanied by the initiation of cell aging [4,5]. According to the theory of free radical aging, accumulated ROS can directly interact with lipids, proteins, and nucleic acids to cause oxidative stress and promote cell aging through a series of signaling pathways, which are widely considered to be one of the core mechanisms mediating skin aging [19,20]. Studies have reported that ultraviolet radiation can lead to an increase in intracellular ROS expression. Oxidative stress induced by ROS accumulation promotes the release of proinflammatory factors from cells, leading to skin inflammation and accelerating oxidative skin damage [40,42].

The effects of 100 μg/mL, 200 μg/mL, 400 μg/mL, and 800 μg/mL peach gum polysaccharide on ROS-induced photoaging in HaCaT cells were investigated. The results are shown in Figure 2B. After UVB irradiation, large amounts of ROS were produced in HaCaT cells (*p* < 0.01, *p* < 0.001), which led to different degrees of apoptosis in cells, indicating that oxidative damage increased and antioxidant capacity was impaired in HaCaT cells exposed to UVB irradiation. In contrast, different concentrations of peach gum polysaccharide and the positive control drug sodium hyaluronate inhibited the production of intracellular ROS in a dose-dependent manner. Compared with the UVB model group, the inhibitory effect of 400 μg/mL and 800 μg/mL peach gum polysaccharide on intracellular ROS production was more obvious. Li et al. used *Sophora flavescens* polysaccharide to treat UVB-irradiated cells and found that high concentrations of this polysaccharide alleviated the high expression of ROS and repaired the oxidative damage caused by UVB radiation [44]. The above experimental results showed that peach gum polysaccharide can inhibit the production of ROS in cells, thereby reducing the level of oxidative stress in cells and playing a protective role in skin photoaging.

### 2.6. Effect of Peach Gum Polysaccharide on the Production of Antioxidant Factors in HaCaT Cells

After ultraviolet radiation damages the skin, large amounts of ROS are produced, which causes oxidative stress in the body and destroys cellular DNA, lipids, and protein components. Because normal skin is rich in antioxidant enzymes and nonenzymatic antioxidants, the production of reactive oxygen species can be prevented during the metabolic processes of normal cells. In contrast, excessive exposure to ultraviolet radiation will deplete or even exceed the supply of the skin’s own antioxidants, leading to oxidative stress [4,45]. Studies have shown that antioxidant enzymes protect skin from UVB-induced photodamage by scavenging ROS [46]. As one of the important antioxidant enzymes in the body, the content of superoxide dismutase (SOD) can indirectly reflect the ability of the body to scavenge ROS. Moreover, the content of catalase (CAT) reflects the level of peroxidation in the body and indirectly reflects the degree of damage to intracellular antioxidant capacity. The results in Figure 2C–F show that the levels of CAT and SOD in the UVB model group were significantly lower than those in the control group (*p* < 0.01, *p* < 0.001), indicating that the cells had increased oxidative damage and impaired antioxidant capacity after UVB irradiation. In contrast, the levels of CAT and SOD in the positive control group and peach gum polysaccharide group were significantly increased (*p* < 0.001, *p* < 0.05), indicating that sodium hyaluronate and peach gum polysaccharide could significantly improve these two phenomena in vitro. In addition, compared with the UVB model group, peach gum polysaccharide at 400 μg/mL and 800 μg/mL significantly reduced the intracellular ROS content and increased intracellular SOD activity (*p* < 0.05) in a dose-dependent manner (Figure 2B,C). Specifically, compared with the UVB model group, the improvement in CAT levels in cells treated with 400 μg/mL and 800 μg/mL peach gum polysaccharide increased 1.6 times and 2.4 times (Figure 2F), respectively, and the improvement of SOD levels in cells treated with 400 μg/mL and 800 μg/mL peach gum polysaccharide increased 1.1 times and 1.2 times (Figure 2C), respectively. The above results showed that peach gum polysaccharide could partially restore the activities of superoxide dismutase and catalase in a photoaged HaCaT cell model and that peach gum polysaccharide reduces intracellular peroxidation and maintains the balance between oxidation and antioxidation in HaCaT cells.

### 2.7. Peach Gum Polysaccharide Reduced UVB-Induced Matrix Metalloproteinase Expression

Skin wrinkles caused by repeated exposure to ultraviolet irradiation are mainly due to damage to skin connective tissue [8]. Proteolytic enzymes, such as matrix metalloproteinases, are a class of proteases produced by epidermal keratinocytes or fibroblasts that mediate endoplasmic reticulum ECM remodeling. Matrix metalloproteinases (MMPs), as collagen degradation enzymes, are produced during skin photoaging, and ultraviolet radiation induces high expression of MMP-1 and MMP-3 [47]. MMP-1 is the most important enzyme that degrades ECM components and can destroy the normal structure of collagen fibers and elastic fibers. MMP-3 is a matrix collagenase that degrades denatured collagen. Therefore, the ability of peach gum polysaccharide to regulate matrix metalloproteinase expression in skin photoaging was determined by using the UVB-irradiated HaCaT cell model. The results are shown in Figure 2D–E. Compared with the control group, the expression of MMP-1 and MMP-3 in HaCaT cells in the UVB model group was significantly increased. In contrast, after sodium hyaluronate intervention, compared with the model group, the expression levels of MMP-1 and MMP-3 in HaCaT cells were reduced 3.7-fold and 1.5-fold, respectively. In addition, peach gum polysaccharide also showed a significant effect on reducing MMP-1 and MMP-3 contents. At concentrations of 200 μg/mL, 400 μg/mL, and 800 μg/mL, the expression levels of MMP-1 and MMP-3 in HaCaT cells were significantly inhibited and showed a significant dose-dependent effect. These data show that peach gum polysaccharide can reduce collagen loss by regulating the expression of matrix metalloproteinases in the UVB irradiation HaCaT cell model.

### 2.8. Peach Gum Polysaccharide Alleviates UVB-Induced Skin Photoaging Damage In Vivo

**Figure 3:**
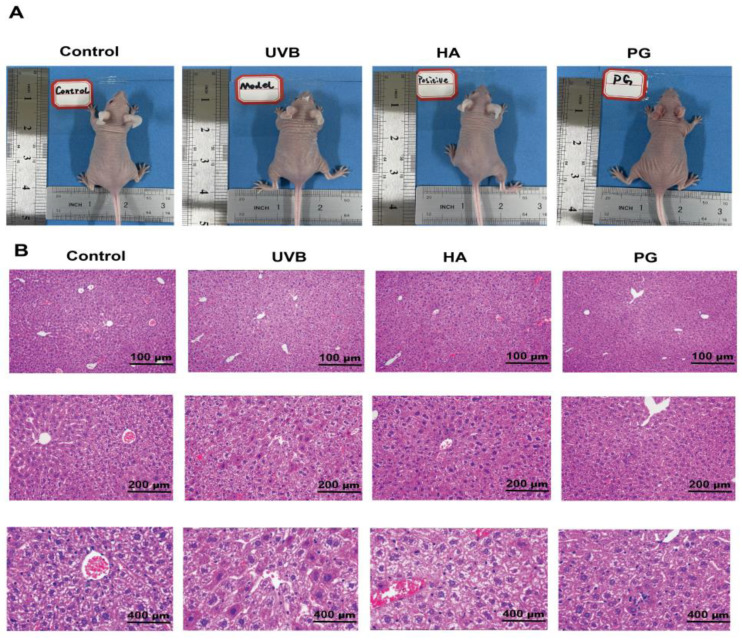
Peach gum polysaccharide alleviates UVB-induced photoaging in mice. (**A**) The dorsal skin of the mice in all groups was taken for phenotype analysis. (**B**) H&E-stained histological images of liver tissues from all groups with different perspectives. Data are expressed as the mean ± SEM from six independent parallel experiments (*n* = 8).

**Figure 4:**
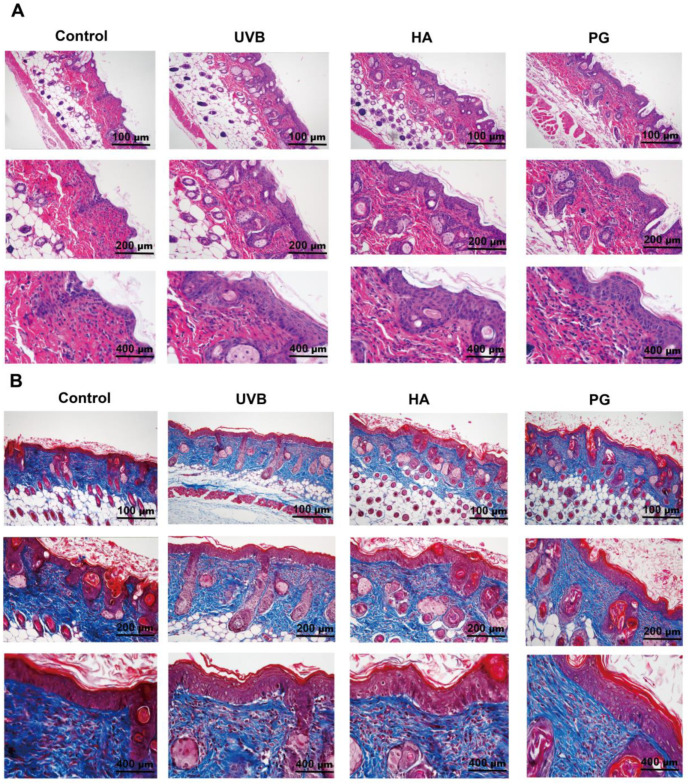
Peach gum polysaccharide is involved in relieving the UVB-induced photoaging in mouse skins. (**A**) H&E-stained histological images of skin tissues from all groups with different perspectives. (**B**) Masson-stained histological images of skin tissues from all groups with different perspectives. Data are expressed as the mean ± SEM from six independent parallel experiments (*n* = 8).

The UVB radiation present in sunlight can penetrate the dermis, with some UVB radiation even reaching the subcutaneous layer, which is considered to be the main factor leading to skin photoaging [26,38]. To study the effect of peach gum polysaccharide on UVB-induced photoaging in vivo, we used hairless mice to construct a photoaging mouse model and then assessed wrinkle formation. The results in Figure 3 indicate that repeated UVB irradiation could lead to changes in skin structure. According to the results of H&E staining, the skins of the mice in the control group showed a relatively complete structure (Figure 4A). However, the skin structure of mice changed after UVB irradiation. These changes included the loss of skin elasticity, severe skin keratinization, increased erythema wrinkles, increased epidermal thickness, and the disappearance of the boundary between the epidermis and dermis. Interestingly, compared with the UVB model group, sodium hyaluronate and peach gum polysaccharide treatment improved the average length and depth of wrinkles and restored normal skin conditions in mouse dorsal skin (Figure 3A). Previous studies have shown that UVB exposure promotes skin dehydration and water loss, resulting in significant dryness and wrinkles [39]. In our study, we found that compared with the UVB model group, different doses of peach gum polysaccharide significantly reduced the thickness of the epidermis and dermis and relieved skin keratinization and wrinkles in vivo. In conclusion, peach gum polysaccharide can reduce the damage caused by UVB radiation on skin photoaging in mice with 200 mg/kg (Figure 4B). Studies have shown that peach gum polysaccharide at a dose of 250 mg/kg/d had a significant inhibitory effect on DSS-induced acute colitis in the animal models, and no toxic side effects on mouse organs [30]. Pathological staining of the liver tissue showed that the liver tissue was intact after intragastric administration of peach gum polysaccharide, indicating that peach gum polysaccharide had no toxicity to the liver (Figure 3B).

### 2.9. Peach Gum Polysaccharide Alleviated UVB-Induced Skin Photoaging In Vivo by Regulating MMPs and Oxidative Factors

**Figure 5:**
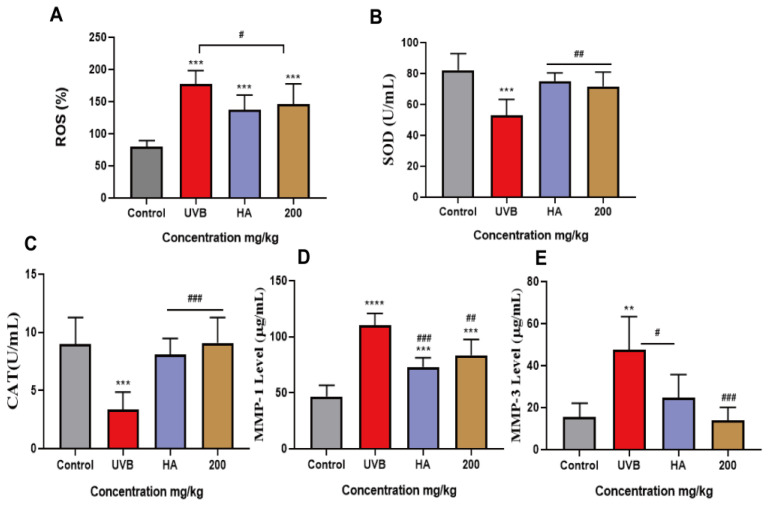
Restoration of antioxidant activity by peach gum polysaccharide. (**A**) ROS level in mouse serum after UVB irradiation. (**B**) SOD level in mouse serum after UVB irradiation. (**C**) CAT level in mouse serum after UVB irradiation. (**D**) MMP-1 level in mouse serum after UVB irradiation. (**E**) MMP-3 level in mouse serum after UVB irradiation. Data are expressed as the mean ± SEM from three independent experiments (*n* = 8). ** *p* < 0.01, *** *p* < 0.001, **** *p* < 0.0001 vs. control. **^#^** *p* < 0.05, **^##^** *p* < 0.01, **^###^** *p* < 0.001 vs. UVB group.

Moreover, to study the inhibitory mechanism of peach gum polysaccharide on UVB-induced photoaging in vivo, Masson staining was used to confirm that oxidative stress and matrix metalloproteinases could cause collagen degradation in photoaged experimental model animals. The results showed that compared with the control group, the number of collagen fibers in the UVB model group decreased, the dermal fibers were twisted and disordered, the number of skin hair follicles decreased, and pore closure increased. In contrast, the numbers of collagen fibers in the skins of mice were restored after treatment with sodium hyaluronate and peach gum polysaccharide, and a large area of the collagen in the skin was repaired, indicating that peach gum polysaccharide inhibited UVB-induced collagen degradation in mouse skin. Studies have shown that the expression of matrix metalloproteinases under ultraviolet irradiation is the main mechanism by which collagen is destroyed. MMP-1 and MMP-3 can decompose type I, type II, type III, or type X collagen [8,47]. Therefore, we further used ELISA to determine whether protection provided by peach gum polysaccharide on photoaging is related to the expression of MMPs induced by UVB irradiation. The results in Figure 5D,E show that the contents of MMP-1 and MMP-3 in the skin in the control group were significantly lower than those in the UVB model group (*p* < 0.05, *p* < 0.01), indicating that the in vivo skin photoaging model was successfully constructed. In contrast, the contents of MMP-1 and MMP-3 in the skins of the mice in the sodium hyaluronate group and peach gum polysaccharide group were significantly lower than those in the UVB model group (*p* < 0.05, *p* < 0.01). Moreover, compared with the UVB model group, 200 mg/kg peach gum polysaccharide treatment reduced the expression of MMP-1 and MMP-3 by 1.0-fold and 3.4-fold, respectively.

In addition, the moisture content and moisturizing degree of mouse skin improved after treatment with peach gum polysaccharide. The results showed that compared with that in the control group, the level of ROS in the serum of the UVB model group increased significantly (*p* < 0.01). In contrast, the level of ROS in the peach gum polysaccharide group was lower than that in the UVB model group, indicating that peach gum polysaccharide could repair oxidative damage (Figure 5A). In addition, the levels of SOD and CAT in the skin were significantly decreased after UVB irradiation, indicating that UVB irradiation caused skin photoaging damage in mice and impaired the skin’s antioxidant capacity. The levels of SOD and CAT in the positive control group and peach gum polysaccharide group were significantly increased (*p* < 0.001, *p* < 0.05), indicating that sodium hyaluronate and peach gum polysaccharide could significantly improve these two indicators in a dose-dependent manner. Compared with the UVB model group, the CAT and SOD levels in the skins of mice treated with 200 mg/kg peach gum polysaccharide increased by 2.1 times and 1.3 times, respectively (Figure 5B,C). The above results showed that peach gum polysaccharide could restore the activities of superoxide dismutase and catalase in the UVB-induced photoaged mouse model, reduce intracellular peroxidation, and maintain the balance between oxidation and antioxidation in vivo.

## 3. Discussion

In this study, we investigated the protective effect of peach gum polysaccharide on UVB-induced oxidative stress and skin photoaging. We found that in in vivo and in vitro models, the addition of peach gum polysaccharide attenuated UVB-induced apoptosis as well as morphological and pathological changes in skin tissue, including deepened wrinkles, collagen degradation and increased epidermal thickness. In addition, dietary supplementation with peach gum polysaccharide can inhibit UVB-induced oxidative stress and the overexpression of matrix metalloproteinases in HaCaT cells and in a mouse model.

China has the highest yield of peaches in the world. Peach gum is an exudate from the stems or branches of peach trees and one of the main byproducts of the peach industry [26,27,48]. Peach gum polysaccharide is one of the main active components of peach gum, which has many health functions, such as regulating blood glucose, alleviating DSS-induced colitis, and exerting anticancer and antioxidative effects [30,31,49]. Ultraviolet (UV) radiation is harmful to human skin and can cause sunburns, immunosuppression, cancer, and skin photoaging [30]. The common clinical manifestations of skin photoaging include the loss of skin elasticity, epidermal atrophy, pigmentation, and even cell necrosis, apoptosis, or cancer [4,38]. Moreover, skin photoaging not only damages the aesthetic appearance of skin and the psychological quality of life but it is also associated with the development of skin cancer and poses a serious health risk. Up to now, laser therapy and surgical treatment have been effective methods to improve the appearance of photoaging skin. However, due to strong laser penetration, if the energy is not properly controlled, the heat generated in the deep tissue or on the skin’s surface may cause soft tissue burns, and there are certain risks with surgical scrubs and chemical peels [9,10].

A large number of studies have reported that natural plant polysaccharides have a good biological protective effect on skin photoaging damage. For example, after HaCaT cells were treated with UVB irradiation (30 mJ/cm^2^), the addition of Cordyceps polysaccharide was shown to increase the activity of SOD and reduce the content of MDA in the cells, which has a notable effect on antioxidation and free radical scavenging in vitro [50]. In addition, Wang et al. studied the UV protection of seaweed sulfated polysaccharides (HFPS) in HaCaT cells and in zebrafish in vitro and in vivo. The results showed that HFPS significantly reduced the level of reactive oxygen species in HaCaT cells, increased cell viability, and reduced apoptosis in a dose-dependent manner. Moreover, HFPS significantly reduced ROS levels, NO production, and lipid peroxidation in a UVB-irradiated zebrafish model in a dose-dependent manner [51]. These studies show that exploring natural active polysaccharides with few side effects to treat skin photoaging diseases has become a focus in antiphotoaging research. However, there are few studies on the effect of peach gum polysaccharide on skin photoaging. Therefore, this study used peach gum polysaccharide as a raw material to construct in vivo and in vitro photoaging models to explore its repair effects.

Long-term exposure to UVB radiation can lead to an increase in ROS in the skin, which can exceed the balance with enzymatic and nonenzymatic antioxidant defense systems in cells and ultimately lead to cell damage [46]. Some studies have reported that plant polysaccharides with high uronic acid contents have good antioxidant activity because of the high molecular weight of this sugar [52,53]. Moreover, turmeric polysaccharides have been noted to be rich in rhamnose, glucose, galactose, arabinose, xylose, galacturonic acid, and glucuronic acid and have shown strong antioxidant activity in vitro. Turmeric polysaccharides significantly improved the scavenging of DPPH and ABTS with an EC_50_ value of 1.478 ± 0.04 mg/mL [54]. In our study, we reported here that peach gum polysaccharide is rich in uronic acid (Table 2). Moreover, Yao et al. found that the reducing ability of peach gum polysaccharide was 0.812 at a concentration of 100 mg/mL. Hydroxyl radicals are the most active among reactive oxygen species and cause serious damage to biological molecules, while DPPH is a stable free radical. Thus, hydroxyl radicals and DPPH have been widely accepted as test indices to evaluate antioxidants. Once the concentration of peach gum polysaccharide reached 100 mg/mL, the free radical removal effect was enhanced with increasing concentration, and the removal rate of DPPH was 91.70% [55]. Our results also confirmed that UVB radiation can cause excessive levels of ROS to be present in cells. Importantly, peach gum polysaccharide is a uronic acid-rich polysaccharide that also contains glucose, galactose, galacturonic acid, and glucuronic acid. Peach gum polysaccharide pretreatment significantly reduced UVB-induced ROS levels in vivo and in vitro (Figure 2B and Figure 5A). Moreover, this oxidative stress response also reduced the levels of oxidative stress factors such as SOD and CAT (Figure 2 and Figure 5). The results show that UVB radiation reduces the expression level of CAT and downregulates the expression of SOD; however, peach gum polysaccharide can significantly reverse these effects. Moreover, a high dose of peach gum polysaccharide could increase the expression levels of CAT and SOD and repair the oxidative damage caused by UVB radiation. Many plant polysaccharides can promote antioxidant effects in cells exposed to ultraviolet radiation by regulating antioxidant enzymes, including catalase and glutathione peroxidase (GSH-Px) [7].

In addition, long-term ultraviolet radiation exposure can lead to adverse changes in the extracellular matrix and aggravate skin photoaging [26]. When ROS-mediated matrix metalloproteinases such as MMP-1 and MMP-3 are overexpressed, these MMPs degrade the extracellular matrix and skin collagen, especially type I collagen, in skin cells [15,17]. MMP-1 is an interstitial collagenase and the most important enzyme in the process of extracellular matrix degradation. It can destroy the normal structures of collagen fibers and elastic fibers. MMP-3 is a matrix-hydrolyzing protease mainly distributed in the extracellular matrix [14,19,20]. After collagen is lost, the skin will exhibit serious aging phenomena. In addition, after ultraviolet radiation, the self-healing ability of damaged collagen is weakened, resulting in a decrease in the contents of collagen fibers and elastic fibers, which increases the degree of skin relaxation, sagging, and aging [27]. Zhang et al. found that fermented dendrobium polysaccharides could downregulate the activity and relative expression of MMP-1 and MMP-3 in HSF cells [36]. Our experimental results also show that peach gum polysaccharide can reduce the contents of MMP-1 and MMP-3 in HaCaT cells and photoaging animal models under UVB radiation and protect skin collagen in vivo and in vitro (Figure 2D,E, Figure 4 and Figure 5D,E).

At present, peach gum polysaccharide is used in the food and pharmaceutical industries. For example, it has been used to make edible food preservative films and prolong the shelf life of fresh shrimp because of its good antioxidant and antibacterial activities [56,57]. Moreover, because of its dense structure, it is used as a low-cost natural plant adsorbent to adsorb pigments and toxic chemical components [58]. Although research on the antiphotoaging effects of peach gum polysaccharide have certain potential, it is still necessary to further purify and modify its structure in the future or combine it as a carrier for reported strong photoaging substances such as phenols to improve the photoaging activity in vitro and in vivo. Moreover, the specific antiphotoaging molecular mechanism of peach gum polysaccharide needs further study.

## 4. Materials and Methods

### 4.1. Plant Materials and Reagents

Peach gum was prepared by Kunming (Yunnan, China). MTT, penicillin-streptomycin, DMSO, and PBS were purchased from Solarbio Technology Co., Ltd. (Beijing, China) and stored at −20 °C. MMP-1 (MM-0072H2) and MMP-3 (MM-0108H2) ELISA kits were purchased from MeiMian Technology Co., Ltd. (Yancheng, China). Fetal bovine serum and Dulbecco’s modified Eagle’s medium were obtained from Gibco Co., Ltd. (Carlsbad, CA, USA). A reactive oxygen species assay kit (CA1410), superoxide dismutase assay kit (BC0170) and catalase (CAT) assay kit (BC0205) were purchased from Solarbio Technology Co., Ltd. (Beijing, China).

### 4.2. Preparation of Polysaccharides from Peach Gum

Coarse peach gum powder was ground into a superfine powder with an ultrafine pulverizer. The superfine powder (100 g) was refluxed twice (for 3 h each time) with 400 mL of 90% ethanol to remove pigments and alcohol-soluble impurities. The residue was dried at 60 °C and extracted with boiling water (100 °C) at a sample-to-solvent ratio of 1:30 (*w*/*v*) for 2 h. After centrifugation, the aqueous supernatant was concentrated at 60 °C and precipitated with 95% ethanol to obtain a final ethanol concentration of 80% at 4 °C overnight. The precipitates after centrifugation (8000× *g*, 20 min) were dried at room temperature, redissolved in distilled water, and then freeze-dried to obtain the water-soluble peach gum polysaccharide designated PG.

### 4.3. Average Molecular Weight (Mw) Determination

Molecular weights were determined on a high-performance liquid chromatography instrument (Agilent 1260 Infinity Ⅱ MDS, Santa Clara, CA, USA) with a gel filtration chromatography column (PL aquagel-OH Mixed-H; 7.5 × 300 mm). The mobile phase consisted of a 0.1 mol/L sodium nitrate aqueous solution flowing at 1.0 mL/min. All the samples were filtered through a 0.22-µm filter membrane before being analyzed. Detection was carried out at 45 °C with a refractive index detector (Agilent 1260, Santa Clara, CA, USA).

### 4.4. Monosaccharide Composition Analysis

The appropriate amount of the sample was weighed in the hydrolysis tube, and 1 mL of water and 4 mol/L trifluoroacetic acid were added. The sample was hydrolyzed at 110 °C for 5 h and then cooled to room temperature. After that, 0.1 mL of the sample was placed in a 4 mL centrifuge tube and dried at 60 °C for 2 h. NaOH (0.3 mol/L) and methanol solution were added to the centrifuge tube. The solution was incubated in a water bath at 70 °C for 60 min and then cooled to room temperature. Next, 0.3 mol/L HCl, 0.75 mL of water and 1.5 mL of chloroform were added. The supernatant was filtered through a 0.45-µm aqueous membrane and loaded into a sample bottle for use. Analysis was conducted on an HPLC system (Agilent 1200 MDS, Santa Clara, CA, USA) with an analytical Agilent C18 column (4.6 mm × 250 mm × 5 μm). The mobile phases were 0.1 mol/L 0.1 M KH_2_PO_4_ (pH 6.8) and acetonitrile (82:18, *v*/*v*) with elution performed at a flow rate of 1.0 mL/min at 25 °C. The UV detection wavelength was set to 254 nm.

### 4.5. Determination of the Chemical Composition

The content of total sugars in the PG samples was determined using the phenol-sulfuric acid method. The protein content was determined using the BCA method. The contents of uronic acids in the PG samples were determined using the standard carbazole sulfuric acid method.

### 4.6. Cell Line Culture and UVB Irradiation

Normal human keratinocytes (HaCaT cell line) were obtained from the Kunming Cell Bank of the Typical Culture Preservation Committee of the Chinese Academy of Sciences.

Cells were cultured in DMEM-high medium with 10% FBS and 1% antibiotics (penicillin and streptomycin) in a humidified atmosphere with 5% CO_2_ at 37 °C. HaCaT cells were treated with peach gum polysaccharide prior to UVB exposure. After 12 h, the cells were washed with PBS and irradiated with UVB (15 mJ/cm^2^) in a small volume of PBS. Then, the cells were cultured for 24 h, and cell samples were collected to determine the related indicators.

### 4.7. MTT Cell Viability Assay

HaCaT cells were treated with different concentrations of peach gum polysaccharide. Next, the cells were incubated for different lengths of time, and then 5 μg/mL MTT was added to each well. After incubation for an additional 4 h, the medium was replaced with DMSO, and OD values were measured at a wavelength of 490 nm with a microplate reader. The cell inhibition rate was calculated using the following equation:

inhibition rate of keratinocyte cells proliferation = 1 − (OD value of the drug treatment group/OD value of the cell control group) × 100%.

### 4.8. Enzyme-Linked Immunosorbent Assay (ELISA)

For cell samples, HaCaT cells were inoculated in a six-well plate for 24 h and treated with peach gum polysaccharide. Then, the cell supernatant was removed and stored at −80 °C. For mouse blood samples, samples from the heart were collected into a heparinized tube, centrifuged at 3500 rpm for 30 min and stored at −80 °C. For mouse skin samples, dorsal skin tissue was obtained and quickly frozen in liquid nitrogen.

The levels of MMP-1, MMP-3, ROS, SOD, and CAT were investigated by ELISA kits according to the instructions. The colorimetric reaction was developed with a chromogenic reagent and the results were read using a microplate reader.

### 4.9. In Vivo Animal Photoaging Model

To determine the effects on photoaging in vivo, 32 healthy male hairless nude mice (five weeks old, 20 ± 2 g in weight) were provided by Charles River Experimental Animal Technology Co., Ltd. (Beijing China) and maintained in a specific-pathogen-free (SPF) environment. The mice were housed under a 12 h/12 h light/dark cycle and provided standard free laboratory food and water. After 7 days, the mice were randomly divided into 4 groups with 8 animals in each group as follows: control group (without any treatment); UVB model group (UVB irradiation); positive control group (sodium hyaluronate, 100 mg/kg); and peach gum polysaccharide (200 mg/kg).

Mice were exposed to 250 mJ/cm^2^ UVB radiation (WFH-203) every other day for 28 days to induce skin photoaging. The skins of the mice showed erythema, flaking, wrinkles, and other photoaging-related changes, indicating that the skin photoaging mouse model was constructed successfully. After 28 days of the experiment, the mice were euthanized with carbon dioxide, and the dorsal skin tissue was obtained and quickly frozen in liquid nitrogen and formalin for additional analysis. The animal experiment was reviewed and approved by the Yunnan Agricultural University Animal Ethics Committee (approval number YNAU-2020-015).

### 4.10. Skin Tissue Histological Analysis

Skin tissue samples from each group were fixed in 10% formalin. Then, all skin samples were embedded in paraffin, and sections were stained with hematoxylin and eosin. The pathological epidermal conditions were observed under an optical microscope (Olympus, Tokyo, Japan).

### 4.11. Skin Tissue Masson Staining Analysis

Skin tissues were fixed in 10% formalin. Then, sections were embedded in paraffin and stained with Bouin’s trichrome, hematoxylin, phosphomolybdic acid, and aniline blue. Next, the slices were treated with glacial acetic acid solution. The slices were dehydrated and sealed, and an optical microscope (Olympus, Tokyo, Japan) was used to observe the distribution of collagen.

### 4.12. Statistical Analysis

The experimental data were analyzed using GraphPad Prism 7.0. Values are expressed as the mean ± standard error of the mean (SEM). The results were compared using one-way analysis of variance (ANOVA). * *p* < 0.05, ** *p* < 0.01, and *** *p* < 0.001; and **^#^** *p* < 0.05, **^##^** *p* < 0.01, and **^###^** *p* < 0.001 were considered to indicate statistical significance.

## 5. Conclusions

**Figure 6:**
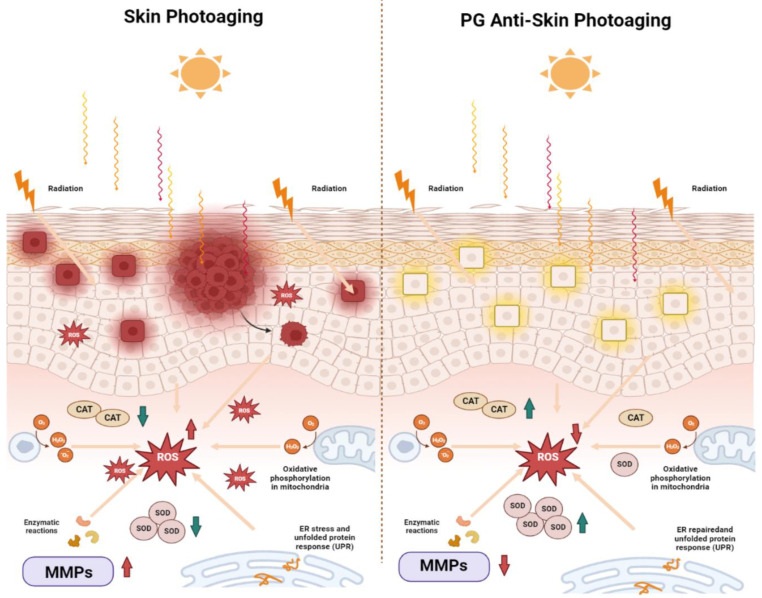
Graphical representation of UVB-induced skin photoaging repair by peach gum polysaccharide.

In summary, in this study, we extracted peach gum polysaccharide with a molecular weight of 4.10 × 10^6^ g/mol from peach gum. In addition, the mechanism by which peach gum polysaccharide protects against UVB-induced skin photoaging in vitro and in vivo was preliminarily revealed. We confirmed that dietary supplementation with peach gum polysaccharide can prevent UVB-induced oxidative stress and photoaging damage in HaCaT cells and in hairless mouse skin. Moreover, peach gum polysaccharide treatment can inhibit the formation of wrinkles by inhibiting the expression of matrix metalloproteinases and increasing and repairing collagen levels. This study provides a theoretical and experimental basis for the development of peach gum polysaccharide as a new antiphotoaging food (Figure 6).

## Figures and Tables

**Table 1:** The chemical composition of peach gum polysaccharide.

Composition	PG
Total sugars	82.34%
Protein	0.18%
Uronic acids (g/100 g)	12.50
M*p* (g/mol)	4.17 × 10^6^
M*n* (g/mol)	4.05 × 10^6^
M*w* (g/mol)	4.10 × 10^6^

**Table 2:** The monosaccharide composition of peach gum polysaccharide.

Monosaccharide	PG (mg/g)
Man	21.96
Rha	7.82
Glu	0.81
Gal	348.69
Xyl	70.63
Ara	362.71
Rib	0.91
Gul A	0.40
Glu A	65.91
Gal A	0.66

## Data Availability

Not applicable.

## Supplementary Materials

The following supporting information can be downloaded at: https://www.mdpi.com/article/10.3390/molecules28104104/s1, Figure S1: Monosaccharide chromatogram analysis Supplementary Materials.
