# Supplementary figures and images for: The Mechanism of Peach Gum Polysaccharide Preventing UVB-Induced Skin Photoaging by Regulating Matrix Metalloproteinanse and Oxidative Factors
Source: Molecules. 2023 May 15;28(10):4104. doi: 10.3390/molecules28104104 (PMC10220684; doi:10.3390/molecules28104104)

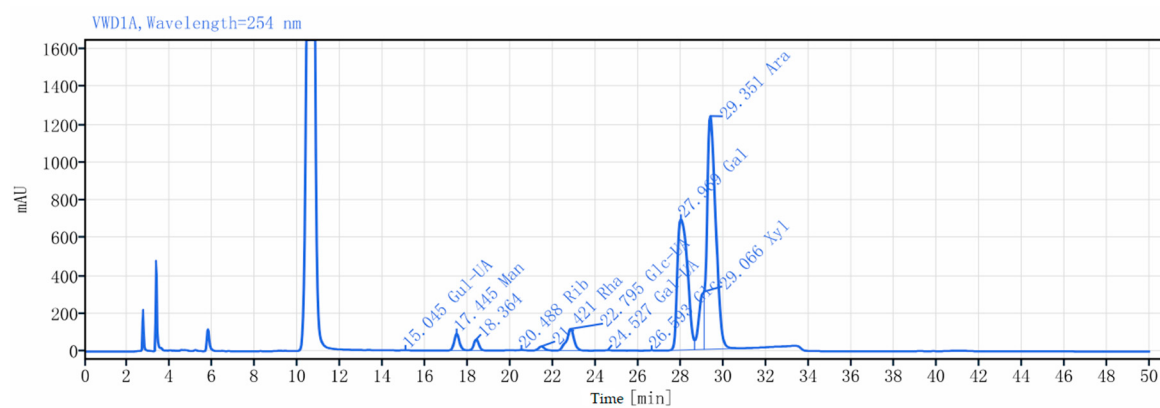

**Figure S1.** Monosaccharide chromatogram analysis supplementary materials.

Supplement: Supplementary file 1 [file molecules-28-04104-s001.zip › molecules-2327162-supplementary.pdf]
